# A Prospective Cohort Study Investigating the Impact of Neutering Bitches Prepubertally or Post-Pubertally on Physical Development

**DOI:** 10.3390/ani13091431

**Published:** 2023-04-22

**Authors:** Rachel Moxon, Sarah L. Freeman, Richard Payne, Jasmine Godfrey-Hunt, Sandra Corr, Gary C. W. England

**Affiliations:** 1 Canine Science, Guide Dogs National Centre, Banbury Road, Leamington Spa CV33 9WF, UK; 2 School of Veterinary Medicine and Science, University of Nottingham, College Road, Sutton Bonington LE12 5RD, UK; 3 School of Biodiversity, One Health and Veterinary Medicine, College of Medical, Veterinary and Life Sciences, University of Glasgow, Bearsden Road, Glasgow G61 1QH, UK

**Keywords:** dog, bitch, neuter, puberty, growth, development

## Abstract

**Simple Summary:**

No previous studies have been identified that have investigated the impact of neutering before or after known puberty on growth and physical development in a large number of bitches. This study was designed to examine data on the physical development, vulval size, and conformation of 306 bitches neutered before (*n* = 155) or after (*n* = 151) puberty. Data were gathered for bitches at six- and 17-months of age using bespoke physical assessment forms and digital photographs of the vulva. Bitches neutered before puberty had significantly greater changes in height and smaller changes in measurements of vulval length and width between six- and 17-months of age than those neutered after puberty. Although not significant, bitches neutered before puberty were taller and heavier with smaller vulval size measurements at 17-months of age. At 17-months of age, significantly more bitches neutered before puberty had vulvas that appeared juvenile and recessed at the physical assessment, and significantly more bitches neutered before puberty had vulvas that appeared ‘recessed/inverted’ on the examination of digital images. The results from this study could suggest that neutering before puberty may be a suitable option for large breed bitches. However, any longer-term health consequences of the differences in physical development seen need to be investigated and better understood before recommendations can be made.

**Abstract:**

No previous large prospective cohort studies have been identified that have investigated the impact of the surgical neutering of bitches before or after known puberty on their growth and physical development. This study was designed to examine the data on physical development, vulval size, and conformation for bitches neutered by ovariohysterectomy before puberty (PPN, *n* = 155) or after puberty (control, *n* = 151) using a prospective cohort study design. Data were gathered at six- and 17-months of age using bespoke physical assessment forms and digital images of the vulva. PPN bitches had greater changes in height measurements (mean difference = 2.039, SEM = 0.334, 91% CI = 1.471 to 2.608, *p* < 0.001) and smaller changes in the measurements of vulval length (mean difference = −0.377, SEM = 0.079, 91% CI = −0.511 to −0.243, *p* < 0.001) and width (mean difference = −0.221, SEM = 0.063, 91% CI = −0.328 to −0.113, *p* < 0.001) between six- and 17-months of age than for the control bitches. Although not significant, the PPN bitches were taller (mean 58.5 vs. 56.6 cm) and heavier (mean 28.3 vs. 27.3 kg) with smaller vulval size measurements (mean vulval length 2.8 vs. 3.2 cm, mean vulval width 1.7 vs. 2.1 cm) at 17-months of age. At 17-months of age, significantly more PPN bitches had vulvas that appeared juvenile (Yates’ Chi-square = 14.834, D.F. = 1, *p* < 0.001) and recessed (Yates’ Chi-square = 7.792, D.F. = 1, *p* = 0.005) at the physical assessment, and significantly more PPN bitches had vulvas that appeared ‘recessed/inverted’ on the examination of digital images (Chi-square = 9.902, D.F. = 1, *p* = 0.002). The results from this study suggest no contraindications to prepubertal ovariohysterectomy for large breed bitches. However, any longer-term health implications of these differences in physical development need to be investigated and better understood prior to recommendations being made.

## 1. Introduction

Sexual maturity in dogs is reached as early as six to seven months of age [1,2,3,4]. While neutering prior to sexual maturity, or ‘early’ neutering, considered to be between 6 and 14 weeks of age [5], is sometimes recommended [6], the literature is inconsistent, making it challenging to decide on the best time to neuter female dogs. Despite many studies investigating the impact of neutering on health [7,8,9,10,11,12,13,14,15,16,17,18,19,20,21,22,23,24], and to a lesser extent, behaviour [9,12,19,25,26,27,28,29,30,31,32], no cohort studies have been identified that investigated the impact of the surgical neutering of bitches before or after known puberty on their growth and physical development.

In dogs, longitudinal skeletal growth is regulated by a combination of genetic, hormonal, environmental, and biomechanical factors [33,34,35]. Oestrogen is associated with inhibition of the growth hormone–insulin-like growth factor axis and closure of the physes at skeletal maturity (potentially by directly influencing growth plate chondrocytes) [34,36]. Early removal of the gonads has been associated with delayed closure of the radial and ulna physes, extended time to reach a growth plateau, and longer radial and ulna bone length in dogs and cats of both sexes [26,37,38,39,40]. The work that is commonly referenced in the dog [26] compared neutered versus entire bitches and bitches neutered at different ages, and identified effects on long bone growth and development of the external genitalia. However, the study included only small numbers and did not consider the timing of neutering in relation to puberty. In contrast, when radial lengths were compared between 11 mixed-breed bitches ovariohysterectomised at 10-weeks of age and 10 bitches that underwent sham surgery [41], no significant differences were identified, and the authors concluded that skeletal development was not impacted by prepubertal gonadectomy. However, final measurements in this study were taken at 24-weeks of age, at which time, their growth may not have reached a stable level.

Oestrogen is also essential for the development of the external genitalia in female dogs [42,43,44]. Oestrogen production prior to the first oestrus causes development of the external reproductive tract and the increase in vulval size [2]; an effect that is maintained by subsequent oestrus cycles. Reduction in oestrogen concentration due to gonadectomy may fail to stimulate normal vulval development, particularly if ovary removal occurs before puberty [42,45]. Salmeri et al. [26] measured the dorsoventral length of the vulvar commissure and reported that subjectively, vulvas in bitches neutered at seven weeks (*n* = 7) or seven months of age (*n* = 4) were smaller than those of entire bitches (*n* = 6), although the numbers of bitches were small and statistical analysis was not performed. This raises the question of whether the length of exposure to ovarian hormones has an effect on vulval development.

Suboptimal vulva conformation is described as a juvenile/infantile or recessed vulva and is associated with increased skin surrounding the vulva, perivulvar skin folds that can trap urine and bacteria leading to bacterial overgrowth, dermatitis and an increased incidence of urogenital disease such as urinary tract infection (UTI), cystitis, and chronic or juvenile vaginitis [4,45,46,47,48,49]. Verstegen-Onclin and Verstegen [42] presented data for 27 bitches with a history of recurrent UTI, vaginitis, and/or perivulvar dermatitis, and on examination, 85% of the bitches had a recessed or hypoplastic vulval appearance and 40% had a perivulvar skin fold. Twenty-five of the bitches were neutered; 21 before or around the time of puberty (mean age at neuter = 4.7 ± 0.3 months) and four after puberty (mean age at neuter = 2.4 ± 0.9 years). The authors proposed that neutering prior to puberty decreases the release of oestrogen and prevents the normal development of external genitalia. However, each of these cases was from a specialist referral hospital with the reason for referral unclear, therefore, the sample was potentially biased due to the study population comprising bitches with known urogenital disease. In contrast, Salmeri et al. [26] and Root-Kustritz [50] suggested that immature vulval development may not cause a clinical problem in otherwise healthy bitches.

Studies on development including general physical characteristics such as height and weight and specific characteristics (e.g., vulval development and abnormalities), and the effect of puberty and surgical neutering on these characteristics in bitches are not well documented in the literature. Such information would be useful to assist with decision making on the optimum time for neutering bitches. Thus, the aim of this study was to compare the measurements of height, weight, body condition score, vulval size, and vulval appearance in bitches neutered either before or after puberty.

## 2. Materials and Methods

### 2.1. Study Design

Using a prospective cohort study design, 306 bitches born in an assistance dog programme between 22 February 2012 and 9 August 2015 were neutered either prepubertally or post-pubertally as previously described [51]. Data were gathered from bitches at six- and 17-months of age to examine their physical development.

### 2.2. Study Setting

Within the assistance dog programme, puppies are placed into volunteer homes with puppy raisers between seven and eight weeks of age. During the puppy raising stage, dogs are neutered before entering formal assistance dog training at approximately 14-months of age. Dogs in the programme are managed under similar conditions and all are fed the same commercially available extruded dry diet from weaning. The study bitches had a physical assessment performed by experienced veterinarians at one of four veterinary practices in the United Kingdom (identified as VP1, VP2, VP3, or VP4) at six-months of age and again at 17-months of age. Physical assessments for bitches that had been withdrawn and rehomed by 17-months of age were performed by a qualified member of the assistance dog organisation’s Health and Well-being team. All assessments were completed within two weeks of the bitches reaching (a) six-months and (b) 17-months of age. For bitches neutered before puberty, the six-month physical assessment was conducted the day prior to neutering. Additionally, the organisation’s electronic health records were examined for bitches with missing assessment forms and missing data on completed assessment forms.

### 2.3. Study Animals

Bitches were allocated into two groups: neutered prepubertally (at six-months of age: PPN, *n* = 155) or post-pubertally (after their first oestrus: control, *n* = 151). Bitches were from five different Labrador/Golden Retriever cross breeds [51]. All 306 bitches were available for physical assessments at six-months of age. By 17-months of age, 56 bitches had been withdrawn from the assistance dog programme. The dog rehoming team were contacted to gather the data from these bitches, with the exception of those rehomed to other working dog organisations. The number of bitches for which data were collected for each assessment was reported.

### 2.4. Variables and Data Sources

#### 2.4.1. Physical Assessments

**Figure 1:**
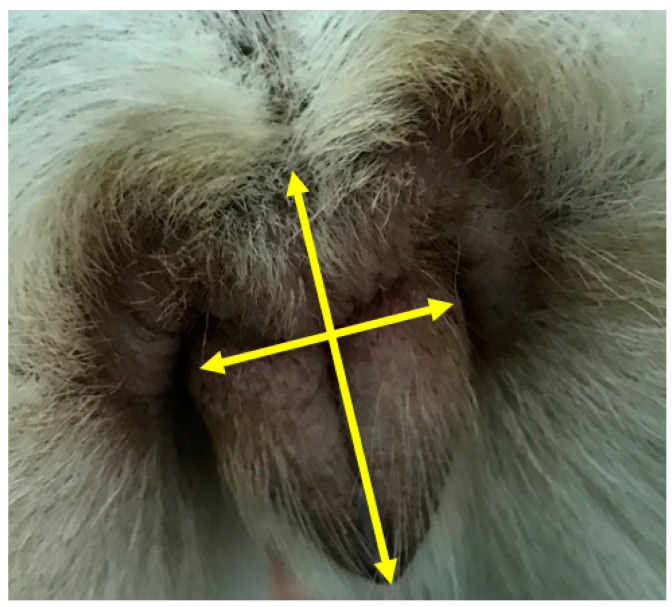
The measurement points (depicted by arrows on the figure) for vulval length (longest arow) and width (shortest arrow) measurements recorded at the six-month and 17-month physical assessments and example of a digital image of a vulva.

Data collection forms in Microsoft Word (See Supplementary Materials S1) were emailed to the Dog Health and Well-being staff member responsible for the bitch when the bitch reached five- or 16-months of age. Forms were completed by the veterinarian or Health and Well-being Specialist assessing the bitch either electronically or in hard copy. Data collected at the six- and 17-month assessments included height at the withers (cm) measured using an equine measuring stick, weight (kg) measured on veterinary practice scales, body condition score (BCS) measured using a 9-point scale (where a score of ‘4’ or ‘5’ represented optimum body condition at six- and 17-months, respectively), vulval measurements (length and width, cm; indicated on Figure 1) measured using a ruler, and descriptive appearance of the vulva. The following six features were assessed and recorded as either ‘0’ [not observed] or ‘1’ [observed]: vulval swelling, perivulvar skin folds, perivulvar dermatitis, vulval discharge, recessed appearance, and juvenile appearance. These scores were later combined to give a cumulative vulva score out of six; bitches with a score of ‘0’ were deemed to have a normal vulval conformation.

At the six-month assessment, the veterinarian also noted any relevant history and clinical signs including evidence of previous or current conditions such as vaginitis or endocrine disease (specific diseases were not specified on the form). Each bitch’s suitability to be neutered at six-months of age (PPN group only) was considered by the veterinarian based on current health and clinical history.

For bitches with missing or incomplete forms for either assessment, electronic health records were examined to determine whether the physical assessment had been completed. Any available height, weight, or BCS data were extracted.

The outcome variables examined at six- and 17-months of age were measurements of bitch height, weight and vulval size, the BCS, responses to questions relating to vulval appearance, and cumulative vulval score. Additionally, the change in measurements between six- and 17-months of age were examined. Confounding variables included breed and the VP performing the assessment as fixed factors, the age at assessment, and the number of days from neutering surgery (six- and 17-month assessments) or the number of days between assessments (for the change in measurements between repeated assessments) as covariates. Confounding variable data were extracted from the organisation’s electronic database or physical assessment forms.

#### 2.4.2. Digital Images of the Vulva

**Figure 2:**
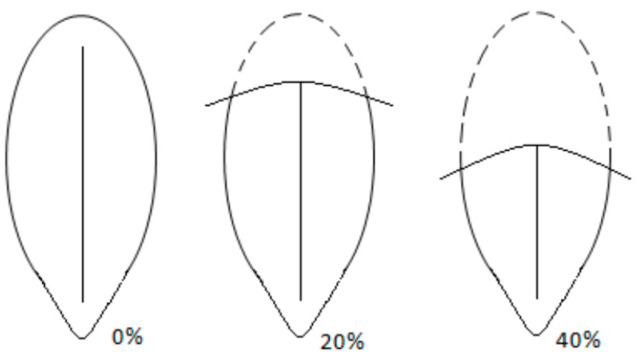
Schematic diagram illustrating 0%, 20%, and 40% dorsal fold percentage cover used during the analysis of digital images of the vulva.

**Figure 3:**
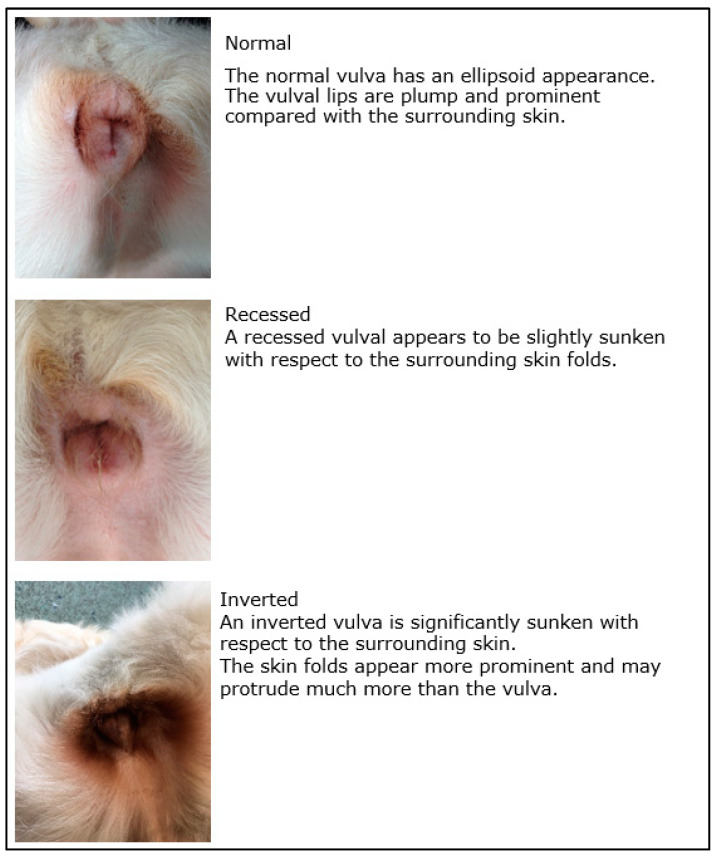
Images and descriptions used to define normal, recessed, and inverted vulval appearance.

A digital photograph of the vulva was taken at the time of the six- and 17-month assessments from a convenient (not fixed) distance (Figure 1). Following a period of training by a senior experienced veterinary reproduction specialist, all vulval images were examined by the same veterinary student who was blinded to bitch age and trial group. A vulval scoring system was devised for four aspects of vulval appearance: presence of vulval discharge, dorsal skin folds, recessed/inverted appearance, and perivulval changes were denoted using ‘0’ (absent) or ‘1’ (present) for each bitch. Vulval discharge (if present) was then categorised as: 1—mucoid, 2—mucoid purulent, 3—purulent, 4—haemorrhagic. Where present, the percentage cover of the vulva by the dorsal skin fold was estimated using 10% intervals (Figure 2). The nature of any perivulval changes were categorised as 1—skin discolouration, 2—hair discolouration, 3—hair and skin discolouration. If any image was of too poor a quality to make a confident assessment, it was recorded as non-diagnostic and excluded from analysis for the corresponding variable. Vulval images were used to describe appearance and assess development at six- and 17-months and were categorised as ‘normal’ or ‘recessed/inverted’ according to Figure 3.

### 2.5. Bias and Study Size

Confounding factors were included in the statistical analysis for bitch height, weight, vulval length, and width. Height and weight can vary with breed, and the effect of different crosses and back crosses on the adult size of Labrador and Golden Retrievers cross bitches is unknown, therefore breed was included in the models. Similarly, age at assessment varied by up to two weeks, which can impact the height and weight. The VP that completed the physical assessment, or whether the assessment was completed by a Health and Well-being Specialist was included due to potential bias in measurement or differences in the calibration of weighing scales. The number of days between neutering surgery and the assessment was also included in the models investigating differences in height, weight, and vulval size at the 17-month assessments.

Study size was determined by the available cohort of bitches that were born during the recruitment period and that were placed with puppy raisers within travelling distance to one of the four national VP for assessments and neutering. G*Power (http://www.gpower.hhu.de/ (accessed on 15 February 2023)) was used to determine the values of alpha for each analysis (see Supplementary Materials S2). The mean (±SEM) and confidence intervals (CI) were reported where appropriate.

### 2.6. Quantitative Variables and Statistical Methods

Data were checked manually for obvious recording errors. Any incorrect dates (identified by assessments appearing to be completed outside of the 2-week period allowed) or weights (unexpectedly low or high for the age) were checked in electronic health records and were corrected where errors were confirmed. Vulval length and width measurements were checked manually, and any cases where length < width were crosschecked with the bitches’ digital images, and corrected if they had been inadvertently transposed (*n* = 4). For statistical analysis, categorical covariates that had less than five bitches were grouped, and one breed group was created that contained all second-generation backcross bitches.

Change in measurements in height at the wither (cm), weight (kg), vulval length (cm), and vulval width (cm) between the six- and 17-month assessments were calculated. For bitches with a calculated change in height that was zero or negative (representing a bitch not growing between six- and 17-months of age, *n* = 19), the data were checked by examining the completed assessment forms to ensure that recording errors were not present. Data errors were excluded from all height analyses and from analyses for other dependent variables where height was included as a covariate. The remaining data were used to examine differences between PPN and the control bitches at six- and 17-months of age, and in the change in measurements between 6-and 17-months of age using univariate general linear models. The dependent factors were bitch height (cm), weight (kg), vulval length (cm), and vulval width (cm) for the six- and 17-month assessments, and were the calculated values for change in height (cm), change in weight (kg), change in vulval length (cm), and change in vulval width (cm). For all models, trial group, breed, and VP were included as fixed factors. For models examining the change in measurements between six- and 17-months of age, VP had five categories: VP1–4 for bitches with physical assessments completed by the same VP at each age, and ‘Different’ for bitches that had assessments completed by different VPs at each age. For the six- and 17-month models, age in days, and for the 17-month models, the number of days between neutering surgery and the assessment, were included as covariates. For models examining the change in measurements between six- and 17-months of age, the number of days between measurements was included as a covariate. Height at the wither was included as a covariate in the general linear models for weight to account for any variation in weight attributable to bitches being taller. Height and weight were included as an interaction term in the general linear models used to examine vulval length and width measurements to control for any variation attributable to bitches being taller and heavier. Standardised residuals were saved and checked for normality (assumptions were met for all general linear models). Pairwise comparisons within the models were used to describe significant differences for the fixed effect variables. General linear models were performed using IBM SPSS Statistics for Windows, version 22 (IBM Corp., Armonk, NY, USA).

A binary logistic regression model using stepwise backward elimination was used to examine the impact of the trial group on BCS while controlling for confounding variables of breed, VP, and body weight. For the six-month assessments, the BCS was grouped as ideal (BCS = 4) and overweight (BCS greater than 4). For the 17-month assessments, the BCS was grouped as ideal (BCS = 5) or under/overweight (BCS less or greater than 5).

Data for visual appearance of the vulva from the assessment forms and digital vulval images and cumulative vulval scores were examined using Chi-square tests. Chi-square with Yates’ continuity correction was used for the analysis of 2 × 2 contingency tables where frequencies were <5. Where frequencies were too small for Chi-square analysis, data were grouped and reported. Sequential Bonferroni correction was applied where multiple testing was conducted to minimise the risk of Type 1 error. Chi-square analyses and binary logistic regression models were conducted using XLStat2016 (Addinsoft, New York, NY, USA).

## 3. Results

### 3.1. Participants

Three hundred and three bitches (152 PPN, 151 control) had completed six-month physical assessment forms. Three PPN bitches did not have completed six-month assessment forms returned; from the examination of electronic health records, all three were neutered at six-months of age at a VP and had a physical assessment noted as completed. All physical assessments at six-months of age were completed by one of the four VPs. All bitches were entire at the time of the six-month assessments. Two hundred and seventy-eight bitches (140 PPN, 138 control) had completed 17-month assessment forms. Twenty-four of these were completed outside of the two-week window and one bitch was still entire at 17 months of age; these were excluded. A total of 253 bitches remained for analysis at the 17-month time point (125 PPN, 128 control). Of these 253 bitches, 251 (123 PPN, 128 control) also had completed six-month physical assessment forms. At the time of the 17-month assessment, 36 of the 253 bitches were withdrawn from the assistance dog programme and 19 bitches had assessments completed by either a Health and Well-being Specialist or a veterinary practice other than one of the four VPs; 234 were completed by one of the four VPs. Nineteen bitches were excluded from all analysis that included height data due to potential errors represented by measurements, suggesting a lack of growth between six- and 17-months of age.

### 3.2. Height

#### 3.2.1. Six-Month

**Figure 4:**
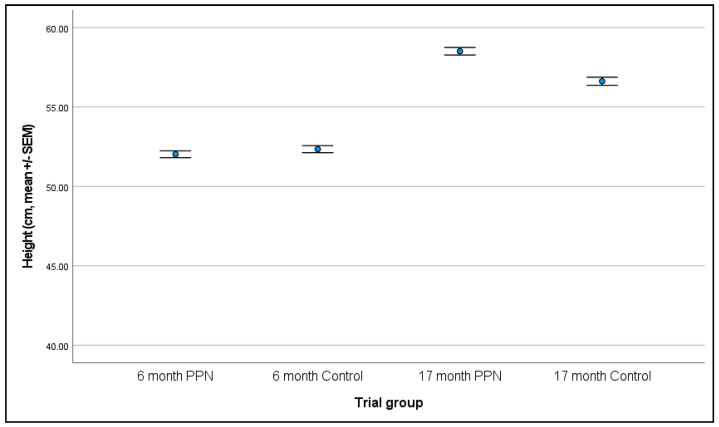
The mean (±SEM) height (cm) for bitches measured at six- and 17-months of age that were neutered prepubertally (PPN) or post-pubertally (control).

Data were available for 282 bitches (146 PPN, 136 control). Bitches measured between 42.0 and 60.0 cm in height (mean 52.2 cm ± 0.2 cm). There was no significant difference in height between the PPN and control bitches at six-months of age (F = 1.941, D.F. = 1, *p* = 0.165; mean height of PPN bitches 52.0 ± 0.2 cm and control bitches 52.3 ± 0.2 cm, Figure 4). Age at assessment (F = 7.396, D.F. = 1, *p* = 0.007) and VP (F = 4.653, D.F. = 3, *p* = 0.003) were significantly associated with bitch height. A one day increase in age was associated with a 0.09 unit increase in height (β = 0.086, SEM = 0.032, 92% CI = 0.030 to 0.142). VP3 had bitches with significantly lower measured heights than VP1 (mean difference = −0.806, SEM = 0.402, 92% CI = −1.512 to −0.100, *p* = 0.046) and VP4 (mean difference = −1.501, SEM = 0.409, 92% CI = −2.220 to −0.782, *p* < 0.001).

#### 3.2.2. 17-Month

Data were available for 229 bitches (119 PPN, 110 control). Bitches measured between 51.0 and 66.0 cm in height (mean 57.6 ± 0.2 cm). There was no significant difference between the PPN and control bitches in height at the 17-month assessment (F = 1.067, D.F. = 1, *p* = 0.303; mean height of PPN bitches 58.5 ± 0.2 cm and control bitches 56.6 ± 0.3 cm; Figure 4). VP was the only factor significantly associated with the height measurement (F = 7.872, D.F. = 4, *p* < 0.001). VP3 had bitches with significantly lower measured heights than all other VPs. Mean differences between VP3 and VP1 was −1.539 (SEM = 0.460, 93% CI = −2.376 to −0.702, *p* < 0.001); VP2 was −1.666 (SEM = 0.497, 93% CI = −2.571 to −0.762, *p* < 0.001); VP4 was –2.311 (SEM = 0.475, 93% CI = −3.175 to −1.447, *p* < 0.001); and ‘Other’ VP was −2.480 (SEM = 0.667, 93% CI = −3.694 to −1.266, *p* < 0.001).

#### 3.2.3. Change in Height between Six- and 17-Month Physical Assessments

**Figure 5:**
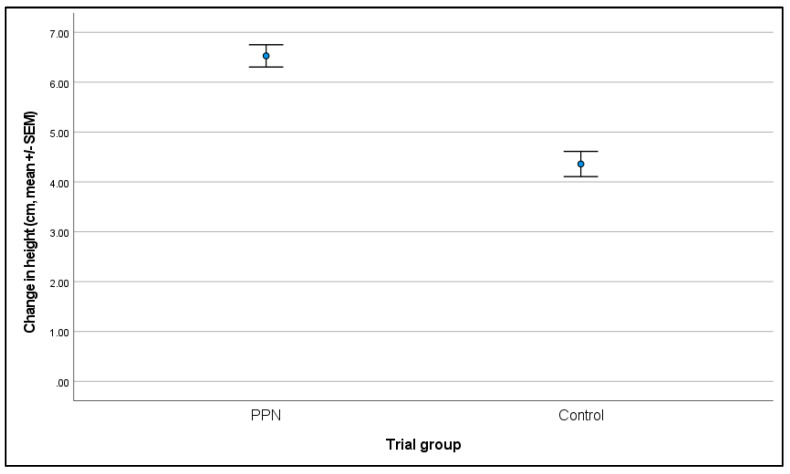
The mean (±SEM) change in height (cm) between measurements at six- and 17-months of age for bitches that were neutered prepubertally or post-pubertally.

Data were available for 225 bitches (115 PPN, 110 control): the mean change in height measurement for PPN bitches was 6.53 ± 0.22 cm and for the control bitches was 4.36 ± 0.25 cm (F = 37.329, D.F. = 1, *p* < 0.001; Figure 5). PPN bitches had a significantly greater change in height measurements than the controls (mean difference = 2.039, SEM = 0.334, 91% CI = 1.471 to 2.608). The number of days between assessments (F = 5.816, D.F. = 1, *p* = 0.017) and VP (F = 2.258, D.F. = 4, *p* = 0.064) also impacted the change in height measurements. A one day increase in days between assessments was associated with a 0.067 unit increase in the change in height measurement (β = 0.067, SEM = 0.028, 91% CI = 0.020 to 0.115). VP3 had a smaller change in height measurements than for all the other VPs. These were significantly smaller than VP2 (mean difference = −1.002, SEM = 0.509, 91% CI = −1.869 to −0.135, *p* = 0.050), VP4 (mean difference = −0.985, SEM = 0.484, 91% CI = −1.810 to −0.160, *p* = 0.043), and for bitches that were measured by ‘Different’ VPs at six- and 17-months of age (mean difference = −1.569, SEM = 0.604, 91% CI = −2.597 to −0.540, *p* = 0.010).

### 3.3. Body Weight

#### 3.3.1. Six-Month

**Figure 6:**
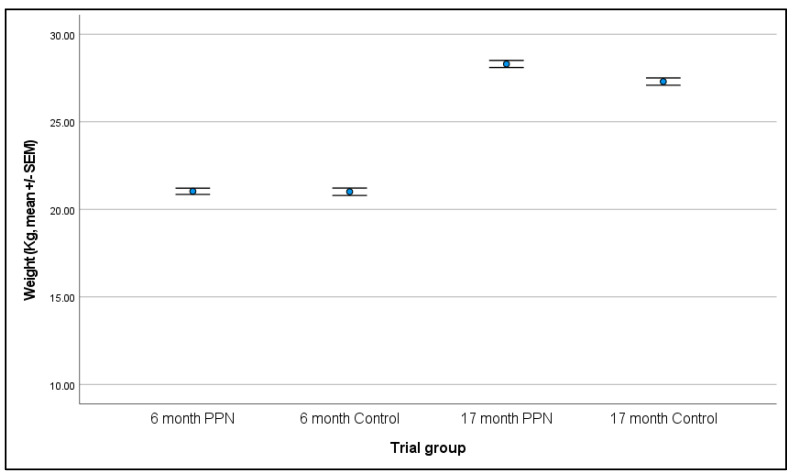
The mean (±SEM) body weight (kg) for bitches measured at six- and 17-months of age that were neutered prepubertally (PPN) or post-pubertally (control).

Data were available for 300 bitches. Bitches weighed between 15.0 and 27.2 kg (mean 21.0 ± 0.2 kg). Data were analysed for 279 bitches (145 PPN, 134 control); two PPN bitches that did not have height data for inclusion as a covariate and 19 bitches with potential errors with height measurement were excluded. There was no significant difference in weight between the PPN and control bitches at six-months of age (F = 0.952, D.F. = 1, *p* = 0.330; mean weight of PPN bitches 21.0 ± 0.2 kg and control bitches 21.0 ± 0.2 kg; Figure 6). Age at assessment (F = 17.221, D.F. = 1, *p* < 0.001), height (F = 52.834, D.F. = 1, *p* < 0.001), and VP (F = 2.688, D.F. = 3, *p* = 0.047) significantly impacted the weight measurements. A one day increase in age was associated with a 0.11 unit increase in weight (β = 0.106, SEM = 0.026, 90% CI = 0.064 to 0.148). A one unit increase in height was associated with a 0.35 unit increase in weight (β = 0.349, SEM = 0.048, 90% CI = 0.270 to 0.429). VP2 had bitches that were significantly heavier at six-months of age than all other VPs (compared to: VP1 mean difference = 0.795, SEM = 0.387, 90% CI = 0.155 to 1.434, *p* = 0.041, VP3 mean difference = 0.808, SEM = 0.352, 90% CI = 0.227 to 1.388, *p* = 0.022, VP4 mean difference = 1.055, SEM = 0.392, 90% CI = 0.407 to 1.702, *p* = 0.008).

#### 3.3.2. 17-Month

All 253 bitches with completed 17-month assessment forms had their body weight data recorded. Bitches weighed between 21.7 and 33.5 kg (mean 27.80 ± 0.1 kg). Five bitches with no height measurement and 19 bitches with potentially incorrect height measurements were excluded from analysis. Data were analysed for 229 bitches (119 PPN, 110 control). There was no significant difference in weight between the PPN and control bitches at 17-months of age (F = 1.289, D.F. = 1, *p* = 0.258; mean weight of PPN bitches 28.3 ± 0.2 kg and control bitches 27.3 ± 0.2 kg; Figure 6). Height (F = 39.050, D.F. = 1, *p* < 0.001) and VP (F = 2.747, D.F. = 4, *p* = 0.029) were significantly associated with body weight at 17-months of age. A one unit increase in height was associated with a 0.35 unit increase in weight (β = 0.345, SEM = 0.055, 91% CI = 0.251 to 0.440). Body weight measurements by ‘Other’ VP were significantly lower than the measurements by all four VPs. Mean differences between ‘Other’ and: VP1 were −1.758 (SEM = 0.566, 91% CI = −2.721 to −0.795, *p* = 0.002); VP2 were −1.328 (SEM = 0.579, 91% CI = −2.314 to −0.342, *p* = 0.023); VP3 were –1.733 (SEM = 0.562, 91% CI = −2.691 to −0.775, *p* = 0.002); VP4 were −1.399 (SEM = 0.571, 91% CI = −2.370 to −0.427, *p* < 0.015).

#### 3.3.3. Change in Body Weight between Six- and 17-Month Physical Assessments

Data were available for 248 bitches (122 PPN, 126 control): the mean change in weight for the PPN bitches was 7.2 ± 0.2 kg and for the control bitches was 6.2 ± 0.2 kg. Bitches with potentially incorrect (*n* = 19) or missing height measurements (*n* = 7) were excluded from the analysis. Changes in weight data were examined for 114 PPN (mean change = 7.2 ± 0.2 kg) and 108 control bitches (mean change = 6.3 ± 0.2 kg), and the trial group had no impact (F = 2.450, D.F. = 1, *p* = 0.119). Breed was not included in the general linear model due to poor model fit.

The number of days between assessments (F = 9.126, D.F. = 1, *p* = 0.003), change in height (F = 8.262, D.F. = 1, *p* = 0.04), and VP (F = 2.640, D.F. = 4, *p* = 0.035) significantly impacted the change in body weight. A one day increase in days between assessments was associated with a 0.077 unit increase in change in weight (β = 0.077, SEM = 0.025, 91% CI = 0.034 to 0.120). A 1-cm increase in change in height was associated with a 0.177 unit increase in the change in weight (β = 0.177, SEM = 0.062, 91% CI = 0.072 to 0.282). VP2 had a smaller change in weight measurements than for all the other VPs, and these were significantly smaller than VP1 (mean difference = −1.350, SEM = 0.479, 91% CI = −2.166 to −0.534, *p* = 0.005), VP3 (mean difference = −1.078, SEM = 0.454, 91% CI = −1.851 to −0.304, *p* = 0.019), and VP4 (mean difference = −1.228, SEM = 0.492, 91% CI = −2.067 to −0.390, *p* = 0.013).

### 3.4. Body Condition Score

#### 3.4.1. Six-Month

Two control bitches did not have a BCS reported at their six-month physical assessment. For the remaining 301 bitches (152 PPN, 149 control), the BCS ranged from 3 to 6 (median = 4; Table 1).

Examination of the BCS data used a binary logistic regression including the data for 297 bitches; two bitches (1 PPN, 1 control) without weight data for inclusion as a covariate, and the two bitches that were BCS 3 (1 PPN, 1 control) were excluded. The model was significant (D.F. = 4, Chi-square = 134.135, *p* < 0.001). Body weight (OR = 1.453, 96% CI = 1.229 to 1.718, *p* < 0.001) and VP influenced whether BCS were ideal (BCS 4) or overweight (BCS > 4) and were retained in the final model. The BCS reported by VP2 were significantly more likely to be reported as higher than ideal than for all the other VPs. The BCS were also significantly more likely to be reported as higher than ideal for VP1 than VP3 and VP4 (see Table S1). The AUC was 0.873. The equation for the best fit model was:

Pred(BCS)=1/(1+exp(−(−9.045+0.374×Weight+3.167×VP2−0.892×VP3−1.276×VP4)))

where BCS = body condition score, VP = veterinary practice (VP1 being the reference practice).

#### 3.4.2. 17-Month

One control bitch did not have a BCS reported at the 17-month physical assessment. For the remaining 252 bitches, BCS at 17-months of age ranged from 4 to 6 (median = 5; Table 2).

Examination of the BCS data used a binary logistic regression including the data for 252 bitches. The model was significant (D.F. = 5, Chi-square = 30.113, *p* < 0.001). Body weight (OR = 0.807, 96% CI = 0.680 to 0.957) and VP influenced whether the BCS were ideal (BCS 5) or under/overweight (BCS 4 or 6) and were retained in the final model. The BCS reported by VP2 were significantly less likely to be ideal (BCS5) than those for VP1, VP3, and VP4. BCS reported by VP4 were significantly more likely to be ideal than those for VP1, VP2, and ‘Other’ (see Table S1). The AUC was 0.771. The equation for the best fit model was:

Pred(BCS)=1/(1+exp(−(7.685−0.215×Weight−1.030×VP2+0.475×VP3+2.346×VP4−0.773−Other)))

where BCS = body condition score, VP = veterinary practice (VP1 being the reference practice).

### 3.5. Vulval Size

#### 3.5.1. Six-Month

**Figure 7:**
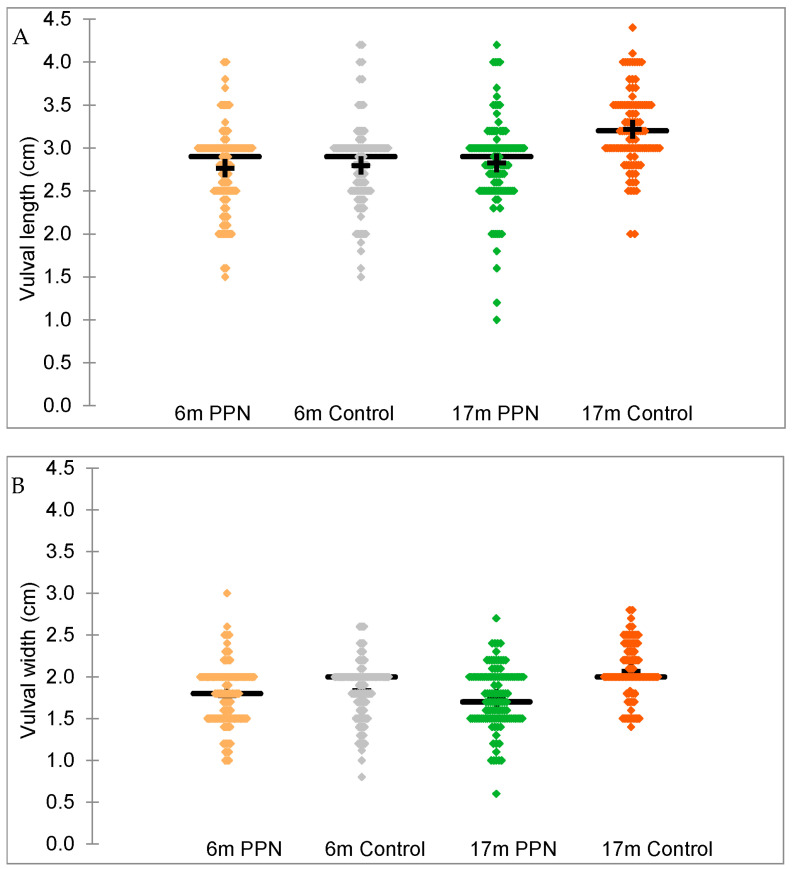
The distribution of vulval length (**A**) and vulval width (**B**) measurements at six- and 17-months of age for bitches neutered before (PPN, *n* = 118) or after (control, *n* = 109) puberty. Graphs show the mean (+) and median (straight line).

Vulval measurements were not reported for one PPN bitch. Six bitches (1 PPN, 5 control) were reported to have a ‘swollen’ vulva appearance at their six-month assessment and were excluded from analysis for vulval size. For the remaining 296 bitches (150 PPN, 146 control), the vulval length ranged from 1.5 to 4.2 cm (mean 2.8 ± 0.03 cm, Figure 7A) and vulval width ranged from 0.8 to 3.0 cm (mean 1.8 ± 0.02 cm Figure 7B). Bitches with no measurements for height (*n* = 1) and weight (*n* = 3) as well as bitches with potential errors in height measurements (*n* = 19) were excluded from the univariate models.

For the remaining 273 bitches (144 PPN, 129 control) mean vulval lengths were 2.8 ± 0.04 cm for the PPN and control bitches and the mean vulval widths were 1.8 ± 0.03 cm for both groups. In the model controlling for the height * weight interaction, there was no significant effect of trial group on the vulval length (F = 0.394, D.F. = 1, *p* = 0.531) or width (F = 2.769, D.F. = 1, *p* = 0.097) measurements. Vulval length was affected by breed (F = 3.808, D.F. = 2, *p* = 0.023), with backcross bitches having significantly smaller vulval lengths than Labrador cross Golden Retriever bitches (mean difference = −0.261, SEM = 0.097, 94% CI = −0.488 to −0.035, *p* = 0.022). The vulval width was affected by age at assessment (F = 7.794, D.F. = 1, *p* = 0.006). A one day increase in age was associated with a 0.01 unit increase in vulval width (β = 0.012, SEM 0.004, 94% CI = 0.004 to 0.020).

#### 3.5.2. 17-Month

Vulval measurements were not reported for six bitches (3 PPN,3 control) at their 17-month physical assessment. For the remaining 247 bitches (122 PPN, 125 control), the vulval length ranged from 1.0 to 4.4 cm (mean 3.0 ± 0.03 cm, Figure 7A) and the vulval width ranged from 0.6 to 3.5 cm (mean 1.9 ± 0.03 cm, Figure 7B). Bitches with no measurements for height (*n* = 1) and with potential errors in height measurements (*n* = 19) were excluded from the univariate models.

For the remaining 227 bitches (118 PPN, 109 control), the mean vulval lengths were 2.8 ± 0.05 cm for PPN and 3.2 ± 0.04 cm for the control bitches and the mean vulval widths were 1.7 ± 0.03 cm for PPN and 2.1 ± 0.03 cm for the control bitches.

In the model controlling for the height * weight interaction, there was no significant effect of trial group on the vulval length (F = 0.011, D.F. = 1, *p* = 0.916) or width (F = 0.175, D.F. = 1, *p* = 0.676) measurements. Vulval length (F = 5.216, D.F. = 4, *p* < 0.001) and width (F = 8.271, D.F. = 4, *p* < 0.001) were affected by VP. Measurements of vulval length were larger from VP1 (mean difference = 0.291, SEM = 0.093, 92% CI = 0.041 to 0.541, *p* = 0.021) and VP2 (mean difference = 0.398, SEM = 0.099, 92% CI = 0.132 to 0.664, *p* < 0.001) than for VP4. Measurements of the vulval length were also larger from VP2 than VP3 (mean difference = 0.255, SEM = 0.092, 92% CI = 0.010 to 0.500, *p* = 0.058). Measurements of the vulval width were larger from VP2 than VP1 (mean difference = 0.338, SEM = 0.069, 92% CI = 0.154 to 0.522, *p* < 0.001), VP4 (mean difference = 0.268, SEM = 0.070, 92% CI = 0.081 to 0.456, *p* = 0.002), and ‘Other’ VP (mean difference = 0.289, SEM = 0.093, 92% CI = 0.040 to 0.537, *p* = 0.021). Measurements from VP3 were larger than from VP1 (mean difference = 0.232, SEM = 0.060, 92% CI = 0.071 to 0.393, *p* = 0.001).

#### 3.5.3. Change in Vulval Size between Six- and 17-Month Physical Assessments

**Figure 8:**
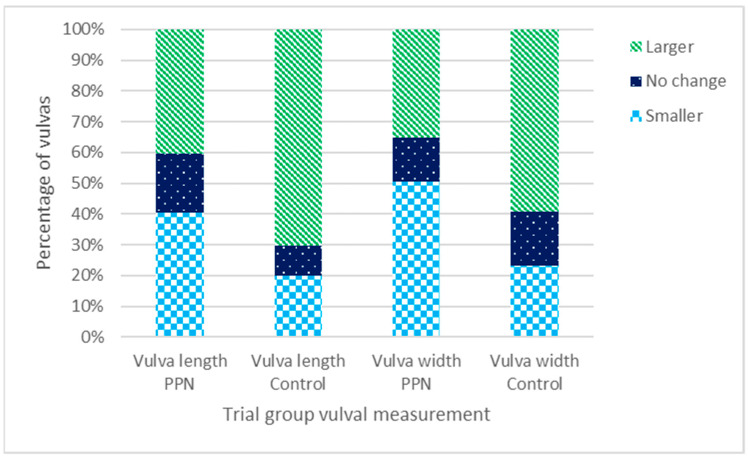
The percentage of bitches neutered before (PPN, *n* = 119) or after (control, *n* = 125) puberty that had vulval length and width measurements that changed to be larger, smaller, or showed no change between their six- and 17-month physical assessments.

Two hundred and forty-four bitches (119 PPN, 125 control) had vulval length and width measurement data available at six- and 17-months of age. The mean change in vulval length and width for the PPN bitches was 0.08 ± 0.05 cm and −0.05 ± 0.04 cm, respectively. The mean change in vulval length and width for the control bitches was 0.39 ± 0.05 cm and 0.19 ± 0.04 cm, respectively (Table 3). Significantly more PPN than control bitch vulvas changed to be smaller and fewer changed to be larger in length (Chi-square = 22.334, D.F. = 2, *p* < 0.001) and width (Chi-square = 20.131, D.F. = 2, *p* < 0.001) between six- and 17-months of age (Figure 8).

**Figure 9:**
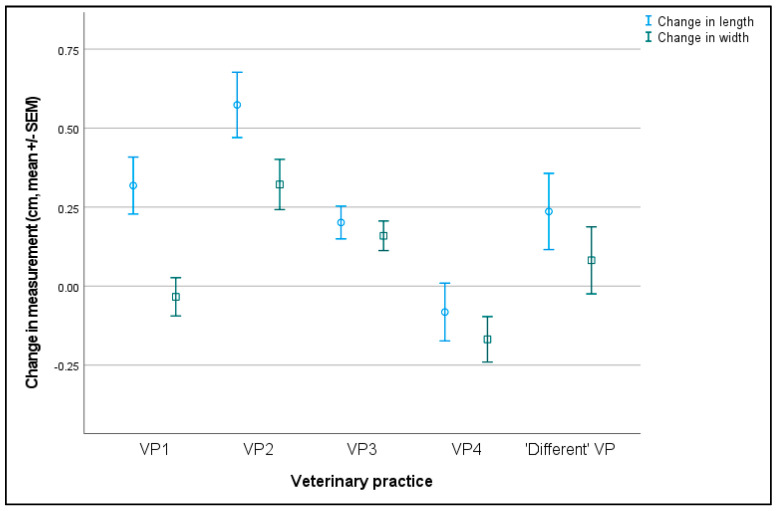
The mean (±SEM) change in the vulval length and width measurements (cm) between six- and 17-months of age for bitches that had physical assessments completed by each veterinary practice (VP) or by ‘Different’ VP for bitches that had assessments completed by different VPs at each age.

Bitches with potentially incorrect or missing height (*n* = 19) or weight (*n* = 5) measurements were excluded from the univariate models. Changes in vulval length and width data were examined for 113 PPN (mean change vulval length = 0.08 ± 0.06 cm, mean change vulval width = −0.05 ± 0.04 cm) and 107 control bitches (mean change vulval length = 0.41 ± 0.05 cm, mean change vulval width = 0.20 ± 0.04 cm). In the model controlling for a height * weight interaction, the mean change in vulval length (F = 22.901, D.F. = 1, *p* < 0.001) and width (F = 12.239, D.F. = 1, *p* < 0.001) was significantly affected by the trial group (Table 4). Changes in both measurements were also affected by VP (vulval length F = 8.005, D.F = 4, *p* < 0.001; vulval width F = 6.932, D.F. = 4, *p* < 0.001; Table 4, Figure 9).

### 3.6. Vulval Appearance

#### 3.6.1. Vulval Appearance at the Physical Assessments

Data on vulval appearance were available for 303 (152 PPN, 151 control) bitches at the six-month stage. The number of bitches with each vulval anomaly was not significantly different between the PPN and control groups following sequential Bonferroni correction (Table 5). Proportionally, more control bitches were reported to have a swollen vulva, vaginal discharge, a recessed or inverted vulva, prominent perivulval skin folds, and perivulval dermatitis. There was no difference in the number of PPN and control bitches with a cumulative vulva score of 0 (106 PPN, 89 control), 1 (28 PPN, 36 control), or 2 and 3 (18 PPN, 26 control) at the six-month assessment (Chi-square = 3.933, D.F. = 2, *p* = 0.140). No bitch had more than three vulval anomalies noted.

Data on vulval appearance were available for 253 bitches (125 PPN, 128 control) at the 17-month stage. Following sequential Bonferroni correction, significantly more PPN than control bitches were reported to have vulvas that appeared juvenile (Yates’ Chi-square = 14.834, D.F. = 1, *p* < 0.001) and recessed (Yates’ Chi-square = 7.792, D.F. = 1, *p* = 0.005; Table 5). No bitches from either trial group had swollen vulvas, and there was no significant difference in the number of bitches reported to have discharge or perivulval folds. Analysis for perivulval dermatitis was not possible due to the small numbers of bitches affected. Significantly more PPN than control bitches had a cumulative vulva score of 1 (18 PPN, 7 control), or 2 and 3 (13 PPN, 3 control) at the 17-month assessment and fewer had a score of 0 (94 PPN, 118 control) (Chi-square = 13.773, D.F. = 2, *p* = 0.001). No bitch had more than three vulval anomalies noted.

#### 3.6.2. Vulval Appearance from Examination of Digital Images

Digital vulval images were captured for 274 bitches (134 PPN, 140 control) at six-months and for 270 bitches (137 PPN, 133 control) at 17-months, although some images were unusable due to poor image quality. The numbers examined for each anomaly are shown in Table 6. At six-months of age, there were no significant differences between the PPN and control bitches in the number of images showing vulval discharge, each category of vulva discharge (excluding ‘haemorrhagic’ due to small n), estimated % dorsal fold coverage grouped as less than 20%, 30%, 40% or greater than 50%, perivulval skin changes, the nature of perivulval skin changes (excluding ‘hair changes only’ due to small n), or ‘recessed/inverted’ appearance (Table 6). Statistical analysis for the presence/absence of dorsal skin folds was not possible due to small numbers of bitches (1 PPN, 0 control) with no dorsal fold present.

**Figure 10:**
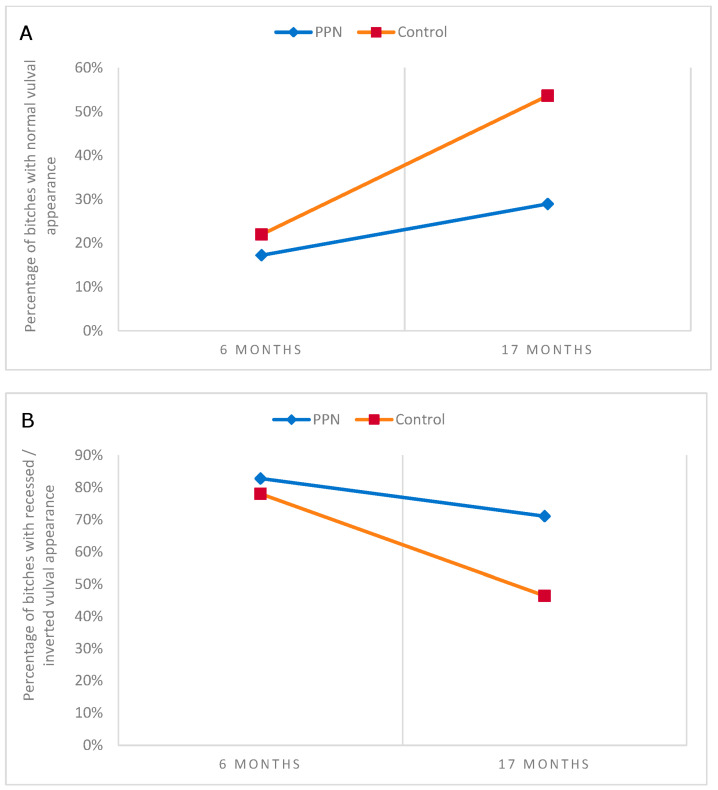
The percentage of bitches neutered before (PPN) or after (control) puberty that had vulval images that were categorised as (**A**) normal or (**B**) recessed/inverted in appearance at six- and at 17-months of age.

Seventy-two PPN (82.8%) and 71 control (78.0%) bitches had vulvas that were classified as abnormal based on being recessed/inverted at six-months of age. At 17-months of age, significantly more PPN (71.1%, *n* = 54) than control bitches (46.3%, *n* = 38) had vulvas that were ‘recessed/inverted’ in appearance (Chi-square = 9.902, D.F. = 1, *p* = 0.002; Figure 10). Ninety-six bitches (46 PPN, 50 Control) had vulvas at both the six- and 17-month assessments that were classified as being ‘recessed/inverted’ or ‘normal’. Ten PPN and 10 control vulvas were ‘normal’ at six months. Of these, three PPN and eight control vulvas remained ‘normal’ at 17-months of age (Yates’ Chi-square = 3.232, D.F. = 1, *p* = 0.072). Thirty-six PPN and 40 control bitch vulvas were ‘recessed/inverted’ at six-months of age. Of these, 10 PPN and 17 control changed to ‘normal’ by 17-months of age (Chi-square = 1.793, D.F. = 1, *p* = 0.181). These differences were not statistically significant.

There was a significant difference in the numbers of PPN and control bitches that had skin discolouration and skin and hair discolouration noted on digital images at 17-months of age (Chi-square = 5.563, D.F. = 1, *p* = 0.018). There were no significant differences in the number of PPN and control bitches that had vulval discharge, each category of vulva discharge (excluding ‘haemorrhagic’ due to small n), estimated % dorsal fold coverage grouped as less than 20%, 30%, 40%, or greater than 50%, or perivulval skin changes (Table 6).

## 4. Discussion

The impact on the physical development of neutering bitches before or after known puberty has not been well-studied. Physical development is an important consideration when deciding when to neuter due to potential consequences on future disease risk. This study presents the first prospective cohort study investigating the impact of neutering before or after puberty on physical development in a large number of bitches. In isolation, the results do not identify any significant contraindications to neutering large-breed bitches prepubertally. However, it is advised that any future consequences for health based on the differences presented here are considered prior to recommendations being made.

Bitches neutered before puberty had significantly greater changes in height and vulval size between six- and 17-months of age than those neutered post-pubertally. Although not significant, bitches neutered prepubertally were also taller and heavier, with vulvas that were smaller at 17 months of age compared to bitches neutered post-pubertally. Interestingly, a dorsal vulval skin fold was apparent in almost all vulval images from bitches at both ages, and percentage cover of the skin fold was reported, which to the authors knowledge is the first time that this feature has been documented in such a large study population. Similar to findings relating to peri and postoperative outcomes [51], veterinary practice was one of the biggest and most consistent influencing factors for height, weight, BCS, and vulva size measurements.

The present study included only female Labrador and Golden Retriever crossbreeds and the findings may not be applicable to other breeds of dog. Similarly, no entire bitches were included for comparison, and therefore no conclusions can be made about whether the physical development observed in bitches neutered pre/post-pubertally differs from that of entire bitches. However, previous studies reporting vulval size [52] suggest that vulval size in bitches neutered after puberty is similar to entire bitches, indicating similar maturation of the external genitalia. Whether vulval size in post-pubertally neutered bitches would subsequently decrease with age is unknown; no later life vulva measurements were obtained. The accuracy of vulval size measurements could have been improved if standardised callipers had been provided to the veterinary practices. Such tools have been used by others [53] and are recommended for future studies.

In the present study, height was measured once, at the time of physical examination, and was subject to measurement variability between the observers and potential inaccuracies. For example, 19 bitches were recorded as having zero or negative change in height between six- and 17-months, and these were subsequently excluded from the analysis. Despite the potential errors, height data were reported due to the importance of considering growth differences between dogs neutered before or after puberty, and the results indicate an impact on growth in a direction that was expected: greater change in height for bitches neutered before puberty with lower levels of oestrogen. Other studies [26,37,38,39,41] have used radiographs to measure the long bone length and identify age at growth plate closure, which would likely provide more accurate ‘height’ data. However, this was not possible within the present study due to ethical constraints.

Two methods of determining vulval appearance were used, with assessments made by veterinarians when examining the bitches, and from digital images of the vulva. While in-person assessment by an experienced veterinarian is useful, the ability to use the digital images enabled one person trained in assessment and blinded to the study group to examine all images, significantly reducing the risk of bias.

There were also effects of VP in almost all analyses. The effects of VP on height were relatively consistent, but were less consistent for the vulva measurements. This highlights the importance of limiting the individuals making measurements, and otherwise controlling for this confounder in analysis. While this does not compromise the findings related to the trial group as VP was included as a covariate in the models, the reasons for the differences warrant further consideration, especially when using multiple veterinary practices to manage a large population of dogs or for future research.

Many authors seem to suggest an impact of early neutering on bitch growth [12,16,18,40,50,54,55,56], and the assertion that this is due to an absence of oestrogen makes sense due to the association of oestrogen with the inhibition of the growth hormone–insulin-like growth factor axis and closure of the physes at skeletal maturity [34,36]. However, most of these are review papers and they reference the one study in dogs by Salmeri et al. [26], which included dogs neutered at seven-weeks of age (seven male, seven female), seven-months of age (four male, four female), and entire dogs (four male, six female) as well as the literature relating to cats [37,39]. Of the two studies that examined long bone length in surgically neutered bitches, both reported increased bone length following early neutering, but the differences were only significant in one of the studies for bitches neutered at seven-weeks of age [26]. For the other, no significant differences were identified, and the authors suggested that there were no skeletal developmental implications of prepubertal gonadectomy [41]. Both of these studies included small numbers of bitches with no consideration of power analysis or adjustment of alpha for the sample size. Only Salmeri et al. [26] considered bitches neutered at different ages; the study by Sontas and Ekici [41] included bitches that were all neutered or underwent sham surgeries at 10 weeks of age and measured the final radial length at six-months of age. Therefore, there are issues with the study design and methodology that should be considered when interpreting these findings.

Our study showed that bitches neutered before puberty were taller (mean 58.5 vs. 56.6 cm) and heavier (mean 28.3 vs. 27.3 kg) by 17-months of age than bitches neutered after puberty, although the differences were not significant. However, the change in height between timepoints did differ significantly by trial group (PPN = 6.5 cm, control = 4.4 cm). Bitches neutered prior to puberty may have an extended growth period, reduced physeal closure, and consequently longer bones and increased height, in agreement with Salmeri et al. [26]. However, Salmeri et al. [26] failed to include whether the bitches had undergone puberty, making direct comparison to the present study difficult. Radiography and measurement of long bone length was not undertaken in the present study. A more accurate assessment of growth and the determination of age at growth plate closure would have been possible from radiographs of the long bones, as described by Salmeri et al. [26]. However, by 17-months of age physeal growth plates would be expected to be closed in all but giant dog breeds [3,57,58]. Therefore, our measurements at 17-months, in contrast to the findings of Sontas and Ekici [41], are likely to be representative of adult height.

The lack of significant findings when comparing height measurements at 17-months for bitches neutered before and after puberty in the present study may be explained by the timing of neutering. The major growth in dogs occurs between three- and six-months of age [58], therefore bitches in both groups were neutered after this period. It is possible that growth plate closure was delayed in bitches neutered before puberty and that this influenced the greater change in height measurements, but that neutering at six-months of age, after the period of major growth had ended, prevented greater differences in height being observed. Salmeri et al. [26] neutered bitches at seven-weeks and seven-months of age, and therefore compared bitches neutered before and after the major growth phase, which could have caused greater differences in the length of the long bones. Indeed, Salmeri et al. [26] only reported significantly greater radial lengths in bitches neutered at seven-weeks (18.5 cm) compared to bitches neutered at seven-months of age and entire bitches (both 16.6 cm). However, comparisons between the studies are impossible due to the different methods of measurement. Age at assessment significantly impacted the height and weight measurements at six- but not 17-months of age in the present study, as would be expected due to more rapid growth around six-months of age compared to at 17-months of age. When considering growth, time before puberty as well as pubertal status at neutering are important factors to consider.

There are few studies examining the impact of neutering on dog growth and related health complications. Studies have demonstrated an increased risk of musculoskeletal diseases such as hip dysplasia and cranial cruciate ligament rupture with neutering [11,12,16]. Spain et al. [12] suggested that the increased bone length caused by early neutering had secondary effects on joint conformation. Bitches neutered prepubertally in the present study were taller and perhaps had increased bone length compared to post-pubertally neutered bitches and could therefore be at increased risk of musculoskeletal disease. There is no research directly investigating the effect of neutering before compared to after known puberty on hip dysplasia or other musculoskeletal disease; studies that examine the impact of neutering at different ages commonly do not consider or define pubertal status at the time of neutering. Examination of health data for bitches in the present study in later life could provide useful information.

While literature relating to the impact of neutering on vulval conformation and development are rare, some authors have suggested that normal vulva development may be impacted by neutering due to the removal of oestrogen, which is essential for the development of the reproductive tract and external genitalia [2,42,45]. In agreement, our study showed differences in vulval size and appearance at 17-months of age for bitches neutered before or after puberty and the change in vulval size between six- and 17-months was significantly greater for post-pubertally neutered bitches; vulval size in prepubertally neutered bitches was similar to that reported for bitches at six-months of age. In a previous preliminary analysis of the data from the present study presented as an abstract, a significant difference was identified; bitches neutered before puberty were found to have significantly smaller vulvas (length 2.9 cm, width 1.8 cm) than bitches neutered post-pubertally (length 3.2 cm, width 2.1 cm) at 17-months of age [52]. In that study, data from entire bitches were also included and vulval sizes for bitches neutered after puberty were not significantly different to entire pure-bred Labradors and Golden Retrievers and their crosses. The reasons why differences were no longer significant in the present study, despite smaller measurements in vulval length and width for prepubertally neutered bitches, are likely due to the more complex method of statistical analysis including confounding variables, and the removal of bitches with missing confounding variable data and incorrect height measurements for inclusion as covariates from the models. Therefore, the authors suggest that the sample size and statistical methodology of the current study provide a robust analysis.

Vulval size has been measured by other authors and compared for bitches neutered at different ages. Salmeri et al. [26] reported that vulvas were smaller and appeared infantile for bitches neutered at seven-weeks (17.8 mm, *n* = 7) and seven-months of age (16.8 mm, *n* = 4) than in entire bitches (19.8 mm, *n* = 6), although the number of bitches was small and statistical analysis was not performed. Marino et al. [59] reported vulval appearance as juvenile and “sunken” for eight bitches that received repeated GnRH implants to delay puberty starting at 4.5 months of age, although actual measurements of the vulva were not performed. In contrast, Kaya et al. [53] reported no difference in vulval size for 13 bitches that received either a deslorelin acetate or a placebo implant between four- and five-months of age for 40 weeks after insertion. The findings of the present study support the suggestion that vulval development may be impeded by neutering prior to puberty.

Studies investigating the effect of GnRH agonists implanted in small numbers of prepubertal bitches with the aim of delaying puberty present conflicting results regarding the impact on height and vulval development that may relate to the age at first implantation, or the number of repeated implants administered [53,59,60]. Marino et al. [59] recruited 24 prepubertal Sicilian hounds to a study and treated eight with GnRH implants at 4.5-, 9-, and 13.5-months of age and 16 bitches did not receive an implant; eight of these were ovariohysterectomised at 18-months of age and eight at 4.5-months of age. The authors reported that the vulvas of the eight bitches that received repeated GnRH agonist implants remained juvenile in appearance at 18-months of age. They also identified no significant difference in weight measurements between bitches in different groups and they noted that all growth plates were closed on radiographs taken at 15-months of age. However, no radiographs were taken at timepoints throughout the study to identify the specific age at growth plate closure, therefore differences could not be compared. Kaya et al. [53] studied the impact of a GnRH agonist implant in 13 medium-sized crossbreed bitches; five bitches that received a 9.4 mg deslorelin acetate implant, four bitches that received a 4.7 mg deslorelin acetate implant, and four bitches that received a placebo implant. Results suggested that there were no significant differences between the groups in body weight, height at the withers, vulval size, and humeral length. However, growth plate closure determined radiographically was delayed for longer in bitches that received the higher dose 9.4 mg implant (83.5 ± 8.5 weeks of age) compared to bitches with the 4.7 mg implant (73.4 ± 4.5 weeks of age) and those with the placebo implant (60.9 ± 9.9 weeks of age). The authors suggest that incomplete suppression of hormones due to a single treatment may have resulted in the lack of observable differences in most measures, although small groups sizes (*n* ≤5 for each) may have affected the reliability of the results.

Results relating to veterinarian assessment of vulval appearance at the six- and 17-month assessments and examination of digital images of the vulva in the present study confirmed that vulva size and development are impacted by neutering before puberty. By 17-months of age, significantly more pre than post-pubertally neutered bitches had vulvas that were juvenile and recessed based on veterinary examination and more classified as recessed/inverted based on digital image examination. These findings are to be expected due to the earlier removal of oestrogen for bitches neutered before puberty, which may prevent oestrogenic influence on the maturation of the external genitalia [2,42,45]. Such anomalies in appearance are suggested to predispose to urogenital disease [4,45,46]. However, seemingly in contrast to the findings related to vulval development and the potential for urogenital disease later in life, fewer bitches neutered before than after puberty had skin discolouration noted when digital images from 17-months of age were examined (23% vs. 39%) and more had skin and hair discolouration (72% vs. 60%). The reasons for this are not clear, but could relate to the presence of disease such as dermatitis or vaginitis; this could result in greater time spent licking or cleaning the perivulval area [61]. Further investigation of urogenital disease incidence for the bitches in this study later in life would be useful to examine this in more detail.

Dorsal occlusion of the vulva is suggested to be an abnormality in vulval conformation which, if a large percentage of the vulva is covered, can lead to mating difficulties and predispose to disease such as vaginitis and cystitis [61]. In the present study, a dorsal vulva skin fold was observed in almost all bitches on digital images of the vulva at six- and 17-months of age, although by 17-months of age, the percentage coverage reduced to 40% or less for most bitches. The authors propose that some coverage of a dorsal fold is a normal anatomical variant in bitches of these breeds based on the majority of the study population having no other clinical disease. However, the physiological purpose of such a fold is unknown.

## 5. Conclusions

Our results suggest that for Labrador/Golden Retriever crossbreed bitches, neutering before puberty does impact growth and physical development. Changes in height and vulval size were significantly impacted by neutering before puberty, and bitches neutered before puberty were more likely to have vulvas that appeared juvenile, recessed, or inverted at 17-months of age. Although not significantly different, bitches neutered before puberty were taller, heavier, and had smaller vulvas at 17-months of age than those neutered after puberty. While these findings suggest no contraindications for prepubertal ovariohysterectomy, the longer-term health implications of these differences in physical development need to be further investigated and better understood before recommendations can be made.

These findings improve our understanding of the impact on physical development of neutering bitches at specific timepoints in relation to known puberty. As such, they will be of interest to veterinarians, assistance and working dog organisations, and pet owners who have to make decisions about whether or not, and when, to neuter female dogs.

## Figures and Tables

**Table 1:** The number of bitches neutered before (PPN) or after (control) puberty of each body condition score (BCS) at six-months of age. The median BCS for each group was 4.

Variable	BCS 3	BCS 4	BCS 5	BCS 6
Breed				
Golden Retriever cross Labrador (*n* = 200)	2	149	46	3
Backcrosses (*n* = 58)	0	34	17	7
Labrador cross Golden Retriever (*n* = 43)	0	31	12	0
Veterinary practice				
1 (*n* = 65)	1	48	16	0
2 (*n* = 52)	0	6	39	7
3 (*n* = 118)	0	103	12	3
4 (*n* = 66)	1	57	8	0
Trial group				
PPN (*n* = 152)	1	106	44	1
Control (*n* = 149)	1	108	31	9

**Table 2:** The number of bitches neutered before (PPN) or after (control) puberty of each body condition score (BCS) at 17-months of age. The median BCS for each group was 5.

Variable	BCS 4	BCS 5	BCS 6
Breed Golden Retriever cross Labrador (*n* = 171)	6	149	16
Backcrosses (*n* = 49)	0	39	10
Labrador cross Golden Retriever (*n* = 32)	2	25	5
Veterinary practice			
1 (*n* = 52)	4	43	5
2 (*n* = 44)	0	29	15
3 (*n* = 87)	1	78	8
4 (*n* = 50)	0	49	1
Other (*n*=)	3	14	2
Trial group PPN (*n* = 125)	4	105	16
Control (*n* = 127)	4	108	15

**Table 3:** The minimum, maximum, mean, and SEM change in vulval length and width measurements (cm) between six- and 17-months of age for bitches neutered before (PPN) or after (control) puberty.

Statistic	Change in Vulval Length (cm)		Change in Vulval Width (cm)	
	PPN N = 119	Control N = 125	PPN N = 119	Control N = 125
Mean ± SEM	0.08 ± 0.05	0.39 ± 0.05	−0.05 ± 0.04	0.19 ± 0.04
Range	−1.4 to 1.7	−1.0 to 1.9	−1.6 to 1.5	−1.0 to 1.3

**Table 4:** The results from the univariate general linear models used to examine the differences in the change in vulval length and width measurements (cm) between six- and 17-months of age for bitches that were neutered before (PPN) or after (control) puberty. The *p* values and model parameter results shown are for the significant effects of neutering before or after puberty and of veterinary practice (VP). * indicates the referent group.

Vulval Measurement	Comparison	Mean Difference	SEM	91% CI	*p*-Value
Vulval length	PPN vs. Control *	−0.377	0.079	−0.511 to −0.243	<0.001
Vulval width	PPN vs. Control *	−0.221	0.063	−0.328 to −0.113	<0.001
Vulval length	VP3 vs. VP2 *	−0.373	0.111	−0.666 to −0.079	0.005
Vulval length	VP4 vs. VP1 *	−0.397	0.112	−0.693 to −0.102	0.005
Vulval length	VP4 vs. VP2 *	−0.676	0.122	−0.997 to −0.354	<0.001
Vulval length	VP4 vs. VP3 *	−0.303	0.108	−0.588 to −0.019	0.054
Vulval width	VP1 vs. VP2 *	−0.353	0.095	−0.603 to −0.103	0.003
Vulval width	VP4 vs. VP2 *	−0.468	0.098	−0.726 to −0.211	<0.001
Vulval width	VP4 vs. VP3 *	−0.304	0.086	−0.531 to −0.076	0.005

**Table 5:** The frequency of observations of anomalies in vulval appearance recorded by veterinarians at the six-month (*n* = 303: 152 PPN, 151 control) and 17-month (*n* = 253: 125 PPN, 128 control) physical assessments for bitches that were neutered before (PPN) or after (control) puberty. The results for the Chi-square tests are presented; significant results following sequential Bonferroni correction are highlighted in bold.

Reported Anomaly in Vulval Appearance	Six-Month Assessment Observed % (N)	Chi-Square (*p* Value)	17-Month Assessment Observed % (N)	Chi-Square (*p* Value)
Swollen vulva				
PPN group	0.7 (1)	1.551 * (0.213)	0.0 (0)	Analysis not possible
Control group	3.3 (5)		0.0 (0)	
Vaginal discharge				
PPN group	9.2 (14)	4.867 (0.027)	4.8 (6)	0.130 * (0.718)
Control group	17.9 (27)		3.1 (4)	
Juvenile				
PPN group	15.1 (23)	1.389 (0.238)	16.0 (20)	14.834 * **(<0.001)**
Control group	10.6 (16)		1.6 (2)	
Recessed/inverted				
PPN group	14.5 (22)	0.428 (0.513)	13.6 (17)	7.792 * **(0.005)**
Control group	17.2 (26)		3.1 (4)	
Prominent perivulval skin folds				
PPN group	3.3 (5)	1.788 (0.181)	4.8 (6)	1.237 (0.266)
Control group	6.6 (10)		1.6 (2)	
Perivulval dermatitis				
PPN group	0.7 (1)	6.092 * (0.014)	0.0 (0)	Analysis not possible
Control group	6.6 (10)		1.6 (2)	

* Chi-square with Yates’ continuity correction.

**Table 6:** The frequency of observations of anomalies in vulval appearance based on the assessment of digital images of the vulva captured at the six- and 17-month physical assessment for bitches that were neutered before (PPN) or after (control) puberty. The results for the Chi-square tests are presented; significant results following sequential Bonferroni correction are highlighted in bold.

Reported Anomaly in Vulval Appearance	Six-Month PPN % (N)	Six-Month Control % (N)	Chi-Square (*p* Value)	17-Month PPN % (N)	17-Month Control % (N)	Chi-Square (*p* Value)
**Vulval discharge**						
Present on image	49.3 (36)	55.0 (44)	0.494 (0.482)	28.8 (23)	22.6 (21)	0.863 (0.353)
Not present	50.7 (37)	45.0 (36)		71.3 (57)	77.4 (72)	
**Vulval discharge categorisation**						
Mucoid	13.7 (10)	7.5 (6)	2.541 (0.281) *	8.8 (7)	6.5 (6)	4.398 (0.222) *
Mucoid purulent	12.3 (9)	18.8 (15)		6.3 (5)	9.8 (9)	
Purulent	23.3 (17)	27.5 (22)		13.8 (11)	5.4 (5)	
Haemorrhagic	0.0 (0)	1.3 (1)		0.0 (0)	0.0 (0)	
**Dorsal skin folds**						
Present on image	98.9 (88)	100.0 (98)	Analysis not possible	97.8 (89)	99.0 (95)	0.002 (0.963) †
Not present	1.1 (1)	0.0 (0)		2.2 (2)	1.0 (1)	
**Estimated dorsal skin fold coverage**						
0%	1.2 (1)	0.0 (0)	2.500 (0.475) **	2.6 (2)	0.0 (0)	4.089 (0.252) **
10%	1.2 (1)	3.3 (3)		7.8 (6)	10.2 (9)	
20%	26.7 (23)	21.7 (20)		32.5 (25)	35.2 (31)	
30%	24.4 (21)	34.8 (32)		28.6 (22)	37.5 (33)	
40%	29.1 (25)	27.2 (25)		19.5 (15)	13.6 (12)	
50%	16.3 (14)	10.9 (10)		9.1 (7)	3.4 (3)	
60%	1.2 (1)	1.1 (1)		0.0 (0)	0.0 (0)	
70%	0.0 (0)	1.1 (1)		0.0 (0)	0.0 (0)	
**Recessed/inverted appearance**						
Present on image	82.8 (72)	78.0 (71)	0.632 (0.427)	71.1 (54)	46.3 (38)	**9.902 (0.002)**
Not present	17.2 (15)	22.0 (20)		28.9 (22)	53.7 (44)	
**Perivulval skin changes**						
Present on image	98.2 (112)	99.1 (109)	0.001 (0.975) †	96.4 (107)	99.2 (118)	0.967 (0.325) †
Not present	1.8 (2)	0.9 (1)		3.6 (4)	0.8 (1)	
**Nature of perivulval changes**						
Skin discolouration	28.8 (32)	31.8 (34)	0.187 (0.666) ‡	23.4 (26)	38.7 (46)	**5.563 (0.018)** ‡
Hair discolouration	2.7 (3)	2.8 (3)		0.9 (1)	0.8 (1)	
Skin and hair discolouration	69.4 (77)	67.3 (72)		72.1 (80)	59.7 (71)	

* Excluding haemorrhagic. ** Dorsal fold coverage grouped as less than 20%, 30%, 40% or greater than 50%. † Chi-square with Yates’ continuity correction. ‡ Excluding ‘hair discolouration’.

## Data Availability

Restrictions apply to the availability of these data. Data were obtained from Guide Dogs UK and are available from Rachel Moxon with the permission of Guide Dogs.

## Supplementary Materials

The following supporting information can be downloaded at: https://www.mdpi.com/article/10.3390/ani13091431/s1, Supplementary Materials S1: Six- and 17-month assessment forms, Supplementary Materials S2: G*Power determined values of alpha for each analysis S2), Table S1: results tables from the binary logistic regression for the analysis of the body condition score.
