# Supplementary material for: A Prospective Cohort Study Investigating the Impact of Neutering Bitches Prepubertally or Post-Pubertally on Physical Development
Source: Animals (Basel). 2023 Apr 22;13(9):1431. doi: 10.3390/ani13091431 (PMC10177277; doi:10.3390/ani13091431)
Supplement: Supplementary file 1 [file animals-13-01431-s001.zip › Animals Supplementary Materials S1 physical assessment forms.pdf]

Supplementary Materials S1

Six month physical assessment form

To be completed for all bitches allocated to the prepubertal neutering study (trial and control dogs).<br><br>To be completed at **6 months** of age, within the individual timeframe specified by Canine Research. For bitches in the PPN group, this form should be completed prior to surgery.<br><br>To be completed by the Centre Vet.<br><br>Please return completed forms and digital photographs to canine.research@guidedogs.org.uk

**Dog Name:**	
**Computer Number:**	
**Trial Group:**	
**Assessment window:**	
**Date completed:**	

<u>General Physical Assessment</u>

**Height at the withers:**<br>To be measured using the measuring stick provided by Canine Research	cms
**Weight:**	Kg
**BCS:**	1 ☐, 2 ☐, 3 ☐, 4 ☐, 5☐, 6 ☐, 7 ☐, 8 ☐, 9 ☐

For 6-month-old puppies please refer to Guide Dogs' Puppy Body Condition Scoring System.

<u>Physical Appearance of the Vulva</u>

Please take a digital photograph of the vulva – to be emailed with this completed questionnaire to the email address above.

The photograph should be taken in portrait orientation at 90º to the vulva. The vulva should occupy the majority of the photograph – please see the photograph below as an example.

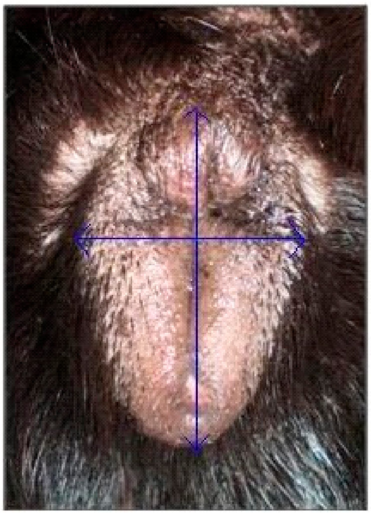

This photograph illustrates the measurement points for the required measurements below:

**Overall length of the vulva:**	cm
**Overall width of the vulva:**	cm

**Does the vulva appear swollen?**	Yes ☐, No ☐
**Is any vaginal discharge present?**	Yes ☐, No ☐
**Does the vulva appear juvenile in appearance? (More so than expected for age)**	Yes ☐, No ☐
**Does the vulva appear recessed/inverted?**	Yes ☐, No ☐
**Are there prominent perivulval skin folds present?**	Yes ☐, No ☐
**Is there any perivulval dermatitis present?**	Yes ☐, No ☐

<u>Clinical History</u>

To be completed from the dog's clinical history and evidence present at the time of the 6-month physical assessment.

**Does the puppy have existing pre-pubertal vaginitis or history of any vulval discharge?**	Yes ☐, No ☐
**Has the puppy already shown signs of oestrus?**	Yes ☐, No ☐
**Is there any evidence of mammary enlargement at the time of the physical assessment?**	Yes ☐, No ☐

**Are there any clinical signs consistent with endocrine disease (including for example symmetrical skin disease, polydipsia, polyuria)?**	Yes ☐, No ☐

17 Month Physical Assessment Form

To be completed for all bitches allocated to the prepubertal neutering study (trial and control dogs).<br><br>To be completed at **17 months** of age.<br><br>To be completed by the Centre Vet or Dog Health and Wellbeing Specialist.<br><br>Please return completed forms and digital photographs to canine.research@guidedogs.org.uk

**Dog Name:**	
**Computer Number:**	
**Trial Group:**	
**Assessment window:**	
**Date completed:**	

General Physical Assessment

**Height at the withers:**<br>To be measured using the measuring stick provided by Canine Research	cms
**Weight:**	Kg
**BCS:**	1 ☐, 2 ☐, 3 ☐, 4 ☐, 5 ☐, 6 ☐, 7 ☐, 8 ☐, 9 ☐

Please refer to Guide Dogs' Adult Dog Body Condition Scoring System.

Physical Appearance of the Vulva

Please take a digital photograph of the vulva – to be emailed with this completed questionnaire to the email address above.

The photograph should be taken in portrait orientation at 90º to the vulva. The vulva should occupy the majority of the photograph – please see the photograph below as an example.

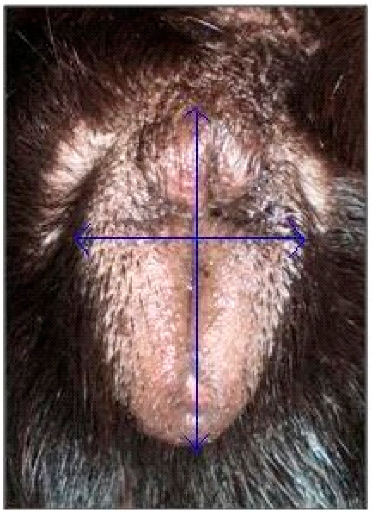

This photograph illustrates the measurement points for the required measurements below:

**Overall length of the vulva:**	cm
**Overall width of the vulva:**	cm

**Does the vulva appear swollen?**	Yes ☐, No ☐
**Is any vaginal discharge present?**	Yes ☐, No ☐
**Does the vulva appear juvenile in appearance?**	Yes ☐, No ☐
**Does the vulva appear recessed/inverted?**	Yes ☐, No ☐
**Are there prominent perivulval skin folds present?**	Yes ☐, No ☐
**Is there any perivulval dermatitis present?**	Yes ☐, No ☐

<u>For dogs that have been withdrawn only (to be used in chapter 9 related to future health):</u>

**Has the dog been diagnosed with any health problems since being rehomed?**	Yes ☐, No ☐
**If 'Yes', please detail**	

**If ‘Yes’, does the owner give consent for Canine Research to contact their veterinary practice to obtain further details if required?**	Yes ☐, No ☐
**If ‘Yes’, please provide contact details for the veterinary practice**	
