# Supplementary material for: A Prospective Cohort Study Investigating the Impact of Neutering Bitches Prepubertally or Post-Pubertally on Physical Development
Source: Animals (Basel). 2023 Apr 22;13(9):1431. doi: 10.3390/ani13091431 (PMC10177277; doi:10.3390/ani13091431)
Supplement: Supplementary file 1 [file animals-13-01431-s001.zip › Animals Supplementary Materials S2 values of alpha.pdf]

# Supplementary Materials S2

## Values of alpha for statistical analysis

For six-month assessments, for height measurements alpha and beta were calculated a priori to give 8% and 5% respectively with an effective sample size of 14 dogs, for weight measurements alpha and beta were calculated a priori to give 10% and 5% respectively with an effective sample size of 58 dogs. For both vulval length and width measurements, alpha and beta were calculated a priori to give 6% and 3% respectively with an effective sample size of 160 (length) and 186 (width) dogs.

For 17-month assessments, for height measurements alpha and beta were calculated a priori to give 7% and 5% respectively with an effective sample size of 14 dogs, for weight measurements alpha and beta were calculated a priori to give 9% and 5% respectively with an effective sample size of 48 dogs. For both vulval length and with measurements, alpha and beta were calculated a priori to give 8% and 4% respectively with effective sample sizes of 94 (length) and 122 (width) dogs.

For change in measurements between six and 17-month assessments, for height measurements alpha and beta were calculated a priori to give 9% and 5% respectively with an effective sample size of 225 dogs, for weight measurements alpha and beta were calculated a priori to give 9% and 5% respectively with an effective sample size of 222 dogs. For both vulval length and with measurements, alpha and beta were calculated a priori to give 9% and 5% respectively with effective sample sizes of 120 dogs.

For analysis of body condition score data, alpha and beta were calculated a priori to give 4% and 5% respectively with effective sample sizes of 270 (6-month) and 175 (17-month) dogs.

For six-month assessments, for visual appearance of the vulva from physical assessment forms, alpha and beta were calculated a priori to give 4% and 5% respectively for all anomalies of appearance, with effective sample sizes between 66 and 304 dogs. For cumulative vulva score, alpha and beta were calculated a priori to give 7% and 9% respectively with an effective sample size of 297 dogs. For appearance of the vulva from examination of digital vulval images, for discharge alpha and beta were calculated a priori to give 9% and 10% respectively with an effective sample size of 157 dogs, for discharge category, alpha and beta were calculated a priori to give 7% and 9% respectively with an effective sample size of 151 dogs, and for all other anomalies, alpha and beta were calculated a priori to give 4% and 5% respectively with effective sample sizes between 21 and 165 dogs.

For 17-month assessments, for visual appearance of the vulva from physical assessment forms, alpha and beta were calculated a priori to give 4% and 5% respectively for all anomalies of appearance, with effective sample sizes between 22 and 42 dogs. For cumulative vulva score, alpha and beta were calculated a priori to give 4% and 5% respectively with an effective sample size of 83 dogs. For appearance of the vulva from examination of digital vulval images, alpha and beta were calculated a priori to give 4% and 5% respectively for all anomalies of appearance with effective sample sizes between 15 and 152.
