# Supplementary material for: A Prospective Cohort Study Investigating the Impact of Neutering Bitches Prepubertally or Post-Pubertally on Physical Development
Source: Animals (Basel). 2023 Apr 22;13(9):1431. doi: 10.3390/ani13091431 (PMC10177277; doi:10.3390/ani13091431)
Supplement: Supplementary file 1 [file animals-13-01431-s001.zip › Animals Supplementary Materials Table S1 BCS binary logistic regression.pdf]

Table S1 results from binary logistic regression for body condition score at (A) six and (B) 17 months of age

A. The odds ratio (OR) and confidence intervals for each predictor variable category for the influence on body condition score at six months of age in comparison to the reference category veterinary practice (VP) (*).

Variable	Value	S.E.	Wald Chi-Square	Pr > Chi²	OR	OR Lower bound (96%)	OR Upper bound (96%)
Intercept	-9.045	1.800	25.258	< 0.0001			
Body weight (Kg)	0.374	0.082	20.955	< 0.0001	1.453	1.229	1.718
Compared to VP1							
*VP1	0.000	0.000					
VP2	3.167	0.550	33.185	< 0.0001	23.737	7.675	73.416
VP3	-0.892	0.420	4.499	0.034	0.410	0.173	0.972
VP4	-1.276	0.529	5.817	0.016	0.279	0.094	0.827
Compared to VP2							
VP1	-3.167	0.550	33.185	< 0.0001	0.042	0.014	0.130
*VP2	0.000	0.000					
VP3	-4.059	0.550	54.529	< 0.0001	0.017	0.006	0.053
VP4	-4.443	0.644	47.582	< 0.0001	0.012	0.003	0.044

B. The odds ratio (OR) and confidence intervals for each predictor variable category for the influence on body condition score at 17 months of age in comparison to the reference category veterinary practice (VP) (*).

Variable	Value	S.E.	Wald Chi-Square	Pr > Chi²	OR	OR Lower bound (96%)	OR Upper bound (96%)
Intercept	7.685	2.465	9.716	0.002			
Body weight (Kg)	-0.215	0.083	6.671	0.010	0.807	0.680	0.957
Compared to VP2							
VP1	1.030	0.500	4.247	0.039	2.800	1.004	7.813
*VP2	0.000	0.000					
VP3	1.505	0.483	9.701	0.002	4.502	1.669	12.142
VP4	3.376	1.067	10.016	0.002	29.256	3.271	261.640
Other	0.257	0.630	0.166	0.684	1.293	0.355	4.711
Compared to V4	-2.346	1.079	4.729	0.030	0.096	0.010	0.878

VP1							
VP2	-3.376	1.067	10.016	0.002	0.034	0.004	0.306
VP3	-1.872	1.075	3.032	0.082	0.154	0.017	1.399
*VP4	0.000	0.000					
Other	-3.119	1.152	7.332	0.007	0.044	0.004	0.471
